# Malignant phyllodes tumor metastasized to the right ventricle: a case report

**DOI:** 10.1186/s40792-015-0121-6

**Published:** 2015-12-05

**Authors:** Fumi Yoshidaya, Naoki Hayashi, Katsuhito Takahashi, Koyu Suzuki, Futoshi Akiyama, Mitsutomi Ishiyama, Yuko Takahashi, Atsushi Yoshida, Hiroshi Yagata, Seigo Nakamura, Hiroko Tsunoda, Hideko Yamauchi

**Affiliations:** Department of Breast Surgical Oncology, St. Luke’s International Hospital, 9-1 Akashi-cho, Chuo-ku, Tokyo 104-8560 Japan; Department of Molecular Medicine and Pathophysiology, Osaka Medical Center for Cancer and Cardiovascular Disease, 1-3-3 Nakamichi, Higashinari-ku, Osaka 537-8511 Japan; Department of Diagnostic Pathology, St. Luke’s International Hospital, 9-1 Akashi-cho, Chuo-ku, Tokyo 104-8560 Japan; Division of Pathology, Cancer institute Hospital of Japanese Foundation for Cancer Research, 3-8-31 Ariake, Koto-ku, Tokyo 135-8550 Japan; Department of Radiology, St. Luke’s International Hospital, 9-1 Akashi-cho, Chuo-ku, Tokyo 104-8560 Japan; Department of Breast Surgical Oncology, Showa University School of Medicine, 1-5-8 Hatanodai, Shinagawa-ku, Tokyo 142-8666 Japan

**Keywords:** Malignant phyllodes tumor, Breast, Cardiac metastasis, Myoepithelial cells, Debulking surgery

## Abstract

Cardiac metastasis of malignant phyllodes tumor is very rare. We herein report a rare case that developed cardiac metastasis from malignant phyllodes tumor. A 38-year-old woman underwent lumpectomy, and the final pathological findings showed the 5-cm malignant phyllodes tumor partially containing 1 cm of squamous cell carcinoma. Four months after the first surgery, a local recurrence of malignant phyllodes tumor and distant metastases to the bone, lung, pulmonary main trunk, and right ventricle were detected. Mass reduction surgery of cardiac metastasis of the malignant phyllodes tumor was performed to avoid sudden death. In immunohistochemical findings, the tumor was suspected to be originated in myoepithelial cells because of the expression of smooth muscle lineage including α-smooth muscle actin and Calponin1 and highly malignant characteristics showing MIB-1 and p53 highly positive with angiogenesis. Further studies are needed to clarify the effective treatment to these tumors.

## Background

Phyllodes tumor is a fibroepithelial neoplasm of the female breast which was classified as benign, borderline malignant, or malignant state, accounting for 0.5 % of all breast tumors [1]. Approximately 50 % of this tumor is benign, and 25 % is borderline [2]. However, it can recur locally in any state [3]. On the other hand, 16 to 30 % of phyllodes tumors were reported to be histologically malignant [4]. Surgical excision is the only effective treatment. Cardiac metastasis of phyllodes tumor is very rare and hard to manage. We herein report a rare case of a patient with a malignant phyllodes tumor that metastasized to the right ventricle and received cardiac surgery.

## Case presentation

A 38-year-old woman visited our hospital with a firm mass in her left breast. The mass had increased in size in a few months. Ultrasounds showed an over 5-cm hypoechoic mass on lower inner quadrant of her left breast. It was diagnosed borderline phyllodes tumor by ultrasound-guided needle biopsy.

**Fig. 1:**
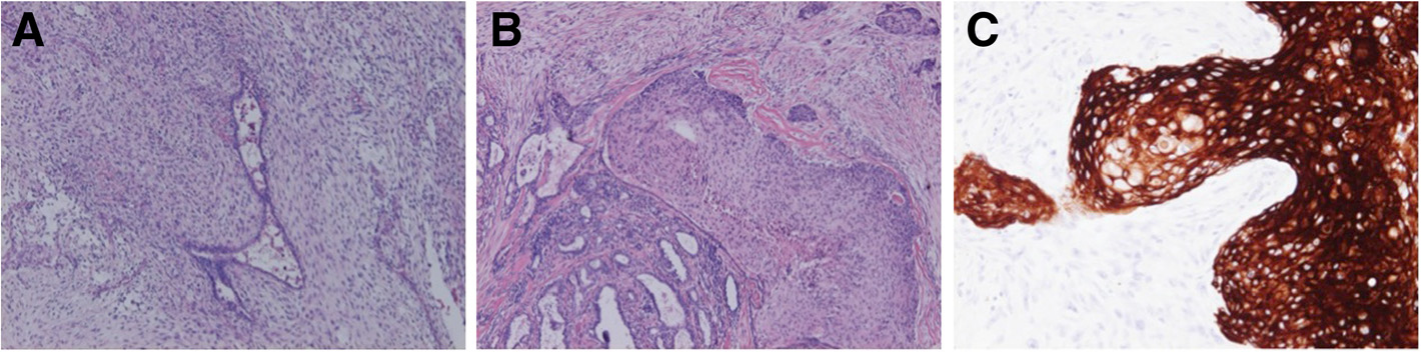
The primary phyllodes tumor in the left breast had leaf-like architecture of epithelial cells and high-grade malignant stromal overgrowth (H&E, ×4 (**a**), magnificent). The tumor partially containing 1.0 × 0.9 cm SCC in the primary site: SCC partially have contact with epithelial cells of phyllodes tumor (H&E, ×4 (**b**), magnificent). AE1/3 was positive on SCC and negative on the phyllodes tumor (×10 (**c**), magnificent). H&E, hematoxylin and eosin; SCC, squamous cell carcinoma

**Fig. 2:**
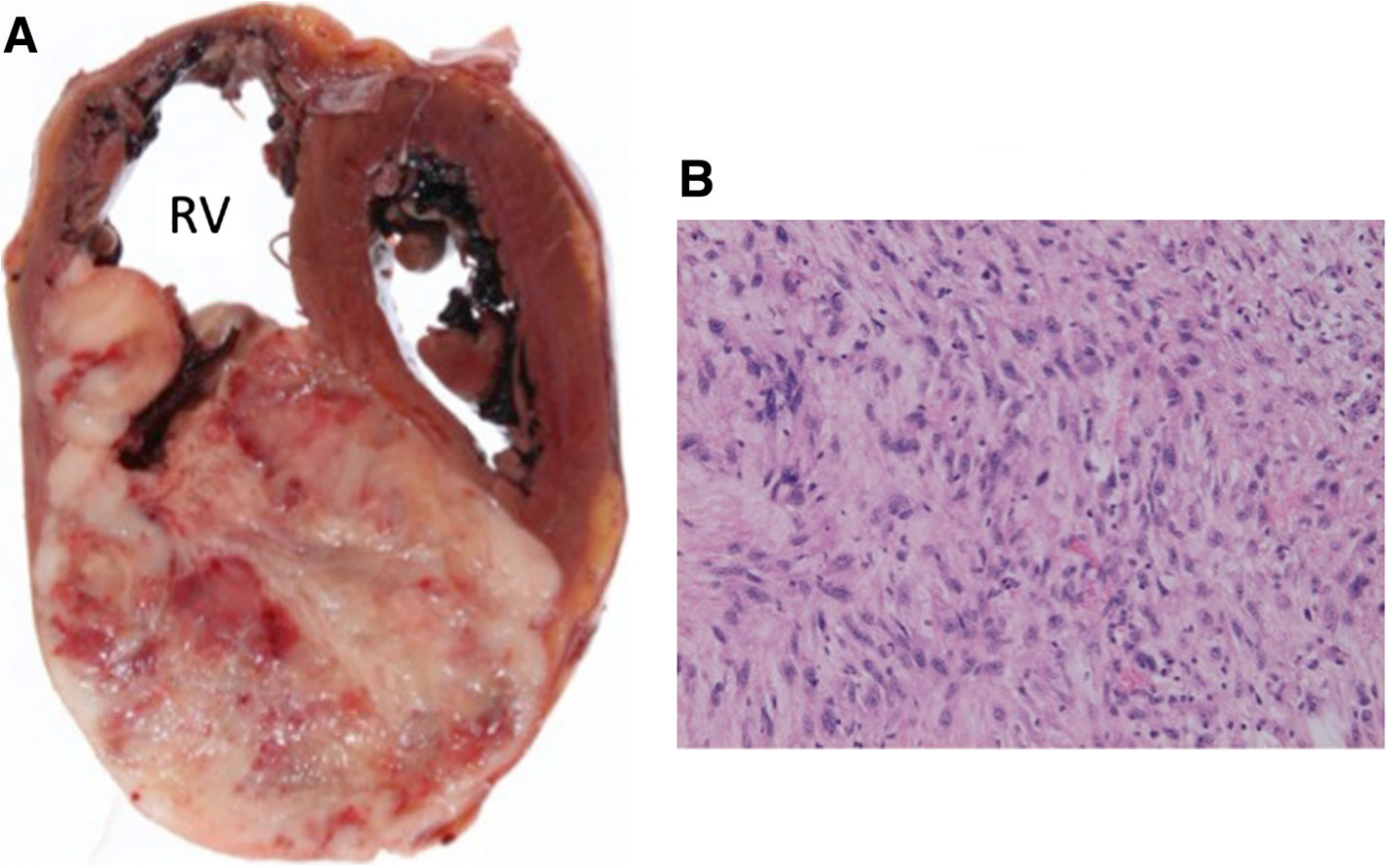
Autopsy specimen of the heart in macroscopic findings (**a**): Cardiac tumor was 10 × 7 × 7 cm, occupied 70 % of the right ventricle. Right ventricle (*RV*) was almost in stenosis. Spindle tumor cells with highly stromal overgrown involving the heart were similar to primary malignant tumor cells in the metastatic site (H&E, ×10 (**b**), magnificent)

The patient underwent lumpectomy. Final pathological findings showed the malignant phyllodes tumor measured 5.0 × 4.7 × .4.0 cm, partially containing 1.0 × 0.9 cm of squamous cell carcinoma (SCC), pT1b N0 M0 stage I, stating that nuclear grade was 2, estrogen receptor was 6, and progesterone receptor was 0 by Allred score, and human epidermal growth factor receptor type2 (HER2) status was negative (Figs. 1 and 2). SCC was not included in the phyllodes tumor but partially contacted with the tumor by epithelial cells.

Because of the margin-positive of phyllodes tumor and the hormone receptor-positive presence of SCC, an additional mastectomy and sentinel node biopsy were performed 1 month after the first surgery. Residual malignant phyllodes tumor was detected but SCC was not found on the surgical specimen. The patient started to receive tamoxifen for the hormone receptor-positive SCC in adjuvant setting.

Four months after the first surgery, she presented in our hospital a 1-month history of a breast tumor on her left chest wall, 1-week history of edema on her face and bilateral legs, dyspnea at rest, back pain, and declining of urine volume, all of which had progressively worsened in a few days. Her consciousness was clear and vital signs were normal, and The Eastern Cooperative Oncology Group performance status was 1. Physical examination revealed edema on bilateral feet and face that indicated congestive heart failure. Chest X-rays showed multiple lung metastasis, enlargement of cardiothoracic ratio (54 %), and protrusion of the left second arch showing pulmonary artery. Computed tomography scan revealed a local recurrence on the left breast, embolism to the right ventricle and pulmonary main trunk, bone destruction (fourth thoracic vertebrae, Th4), and lung metastasis. Transthoracic echocardiography showed a 5.8 × 4.1 × 7.0-cm mass with homogeneous echogenicity that occupied nearly the entire right ventricular (RV) cavity and almost obstructed RV outflow tract (RVOT). It also involved the pulmonary valve and extended to the pulmonary artery. The mass had invaded the RV septum to the endocardium and then grew forward to the RV reaching the pulmonary artery. There were a few evidences of pericardial effusions. We diagnosed a chest wall recurrence of malignant phyllodes tumor and distant metastases to the bone, lung, pulmonary main trunk, and right ventricle.

After multidisciplinary discussion among breast surgeon, medical oncologist, radiologist, cardiologist, and cardiac surgeon, the patient decided to undergo urgent mass reduction surgery releasing the severe stenosis of RVOT to avoid sudden death. The histological finding revealed a metastasis of malignant phyllodes tumor to right ventricle.

She was discharged from the hospital on the 12th postoperative day. However, on the 66th day after the cardiac surgery, she died from heart failure because of re-enlargement of the residual tumor in the right ventricle.

### Pathological findings

**Fig. 3:**
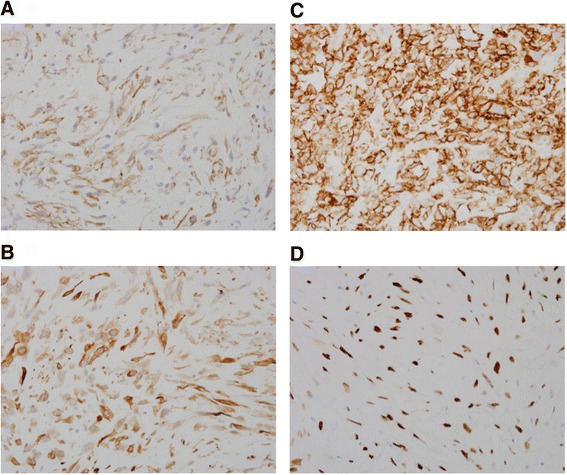
Histologic and immunohistochemical findings of metastatic tumor of the heart: formalin-fixed and paraffin-embedded slices were immunostained, **a** α-smooth muscle actin and **b** Calponin1, **c** tumor suppressor, p53, and **d** VEGFR2 (Flk-1). Bars indicate 100 μm

Autopsy showed the presence of local recurrence and multiple distant metastases to the lung, bone (Th4), diaphragm, and cardiac ventricle of malignant phyllodes tumor (Fig. 3a). Spindle tumor cells involving the heart were similar to primary malignant tumor cells in microscopic findings (Fig. 3b). SCC was not involved in any metastatic sites. In immunohistochemical findings of primary tumor, pan-cytokeratin AE1/AE3 was negative on the phyllodes tumor site while positive on SCC site (Fig. 1d, e). The specimen of metastatic tumor to the right ventricle showed that pan-cytokeratin AE1/AE3 was negative, suggesting to be originated from the malignant phyllodes tumor. Markers suggesting differentiation to smooth muscle, α-smooth muscle actin, and Calponin1 were partially positive. CD44 was positive. MIB-1 was highly positive (≥  70 %), and p53 was strongly overexpressed. Angiogenesis in the tumor was frequently observed as demonstrated by high expression level of vascular endothelial growth factor receptor-2 (VEGFR2) (Flk-1) in tumor vessels.

### Discussion

Once patients with malignant phyllodes tumor developed metastasis, their prognosis is extremely poor. After the development of metastases, the mean overall survival is 30 months [5]. For phyllodes tumors, complete surgical resection with wide margins is the fundamental treatment to prevent metastasis [6,7]. Despite the complete surgical resection, local failure rates range between 20 and 30 % for malignant tumors [8–10].

Approximately 22 % of cases of malignant phyllodes tumor may give rise to hematogenous metastases. In a review of 67 patients who had metastatic phyllodes tumors, Kessinger and colleagues described the frequency of metastasis in each site: lungs (66 %), bones (28 %), heart (9 %), and liver (6 %).

In most cases, the metastases resemble to the sarcomatous component of the primary tumor [11]. The role of any adjuvant therapy, such as chemotherapy, hormonal therapy, and even radiotherapy remains unclear [12].

Cardiac metastasis of malignant phyllodes tumor is very rare. To our knowledge, there are only four cases that underwent cardiac surgery for the metastasis (Table 1) [13–16].

**Table 1:** Cases that underwent cardiac surgery for metastasis

	Age	Pre-operative general status	Surgery	Location of cardiac metastasis	Overall survival from the cardiac operation	Survival duration from initial surgery
Our case	38	Stable	Mass reduction of RV, PA	RV, PA	66 days	3 months
Garg et al. [13]	35	Cardiac shock	Mass reduction of RV	RV	8 days	3 years
Jackson et al. [14]	69	Unstable	Mass reduction of RV, PA	RV, PA	77 days	77 days
Myojin et al. [15]	47	Unstable	Mass reduction of RV, PA Tricuspid valve plasty	RV, PA	15 days	3 months
Nakatsu et al. [16]	69	Stable	Mass reduction of LA	LA	1 year (still alive)	NA

*LA* left atrium, *RV* ventricle, *PA* pulmonary artery, *NA* not applicable

In four out of the five cases including our cases, tumors were metastasized to the right ventricle and one to the left atrium. In these cases, debulking surgery could avoid sudden death, although most cases had unstable condition after the surgery and showed poor prognosis. Surgery to cardiac metastasis has a chance to avoid sudden death and improve the patients’ quality of life and spend better the rest of their lives, regardless of how short and low the quality of their lives are even after the cardiac surgery. The patient strongly hoped to undergo the surgery to have a possibility to prolong her life with her children for a couple of months. After the surgery, her symptom of heart failure was decreased and she and her family could be satisfied to spend the rest of her life at her home.

In the current study, markers that were frequently observed in undifferentiated and high-grade sarcoma were shown in immunohistochemical findings of the specimen. The tumor was suspected to have been originated from myoepithelial cells because of the expression of smooth muscle lineage with Calponin1. Expression of basic calponin has proved not only to smooth muscle cells but also in cells with certain smooth muscle-like phenotypes including myofibroblasts, myoepthelial cells, leiomyosarcomas, synovial sarcomas, and a subset of osteosarcomas [17]. It also showed highly malignant characteristics evidenced by the presence of many cells suggesting high proliferation with highly positive MIB-1 and p53 expression. MIB-1 and p53 expression has been reported as a marker of aggressiveness in many tumors. In the phyllodes tumor, the expression level of p53, tumor angiogenesis, and MIB-1 have been described to reflect the degree of malignancy [18], although these are not correlated with their prognosis.

Belkacémi and colleagues reported that in 159 patients with malignant and borderline phyllodes tumor, radiotherapy significantly decreased local recurrence rate (*p* = 0.02) [20]. In a prospective study of 46 patients with borderline or malignant phyllodes tumor who underwent breast-conserving surgery with adjuvant radiotherapy, none of the 46 patients developed a local recurrence with a median follow-up of 56 months [19]. Although she did not receive irradiation therapy because of her predicted short prognosis, radiotherapy rather than chemotherapy in adjuvant setting may have a good potential for patients with malignant phyllodes tumor after surgical resection for local control and prevention of metastasis.

## Conclusions

We experienced a rare case of malignant phyllodes tumor of the breast with high malignant characteristics that was originated from myoepithelial cells and developed cardiac metastasis shortly after the removal of the primary breast tumor. Our experience indicated that mass reduction surgery of cardiac metastasis of malignant phyllodes tumor might be an option for avoiding sudden death and improving patients’ quality of life, regardless of the extremely poor prognosis. Further large studies are needed to clarify the effective treatment to these tumors.

## Abbreviations

HER2: human epidermal growth factor receptor type2
RV: right ventricle
RVOT: RV outflow tract
SCC: squamous cell carcinoma
